# Associations between leaf developmental stability, variability, canalization, and phenotypic plasticity in *Abutilon theophrasti*

**DOI:** 10.1002/ece3.8845

**Published:** 2022-04-17

**Authors:** Shu Wang, Dao‐Wei Zhou

**Affiliations:** ^1^ 71206 College of Forestry Forest Ecology Research Center Guizhou University Guiyang China; ^2^ Northeast Institute of Geography and Agroecology Chinese Academy of Sciences Changchun China

**Keywords:** aboveground competition, canalization, developmental instability, fluctuating asymmetry, increased density, intraindividual variability, leaf size, phenotypic plasticity

## Abstract

Developmental stability, canalization, and phenotypic plasticity are the most common sources of phenotypic variation, yet comparative studies investigating the relationships between these sources, specifically in plants, are lacking. To investigate the relationships among developmental stability or instability, developmental variability, canalization, and plasticity in plants, we conducted a field experiment with *Abutilon theophrasti*, by subjecting plants to three densities under infertile vs. fertile soil conditions. We measured the leaf width (leaf size) and calculated fluctuating asymmetry (FA), coefficient of variation within and among individuals (CV_intra_ and CV_inter_), and plasticity (PI_rel_) in leaf size at days 30, 50, and 70 of plant growth, to analyze the correlations among these variables in response to density and soil conditions, at each of or across all growth stages. Results showed increased density led to lower leaf FA, CV_intra_, and PI_rel_ and higher CV_inter_ in fertile soil. A positive correlation between FA and PI_rel_ occurred in infertile soil, while correlations between CV_inter_ and PI_rel_ and between CV_inter_ and CV_intra_ were negative at high density and/or in fertile soil, with nonsignificant correlations among them in other cases. Results suggested the complexity of responses of developmental instability, variability, and canalization in leaf size, as well as their relationships, which depend on the strength of stresses. Intense aboveground competition that accelerates the decrease in leaf size (leading to lower plasticity) will be more likely to reduce developmental instability, variability, and canalization in leaf size. Increased developmental instability and intra‐ and interindividual variability should be advantageous and facilitate adaptive plasticity in less stressful conditions; thus, they are more likely to positively correlate with plasticity, whereas developmental stability and canalization with lower developmental variability should be beneficial for stabilizing plant performance in more stressful conditions, where they tend to have more negative correlations with plasticity.

## Graphical abstract

We showed a decrease in developmental instability, intraindividual variation, and plasticity, and an increase in interindividual variation in leaf size with increased density. The insignificant correlations between variables in most cases and negative correlations between interindividual variation and plasticity at high density and/or in fertile soil suggested the complexity of relationships among different mechanisms, and canalization is more likely to facilitate adaptive plastic response under stress.

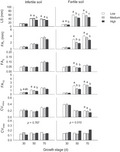

## 1 INTRODUCTION

Phenotypic variation has received increasingly greater attention and become the central topic of ecological evolutionary developmental biology (“eco‐evo‐devo”) (Pfennig, 2016), since the evolutionary significance of phenotypic plasticity was recognized (Bradshaw, 1965). The factors influencing phenotypic variation generally fall into two antagonistic aspects: sources of variation due to genetic, environmental, and developmental effects, and regulatory processes or mechanisms that buffer against variations or improve phenotypic performance. Three regulatory mechanisms being widely investigated are phenotypic plasticity, canalization, and developmental stability (Palmer, 1996; Wagner et al., 1997). Developmental stability, defined as the tendency of traits to resist the effect of developmental errors (Palmer & Strobeck, 1986), is usually measured as fluctuating asymmetry (FA, random deviation from perfect bilateral symmetry) (Møller & Swaddle, 1997). Canalization, or the ability of a genotype to produce consistent phenotypes regardless of environmental and genetic variabilities (Waddington, 1942), uses coefficient of variation (CV) as an index (Woods et al., 1999). By contrast, phenotypic plasticity, or the shift in phenotype due to changes in environments (Schlichting, 1986), is fundamentally evaluated by the difference in a given trait between environments (Valladares et al., 2006). All the three mechanisms of developmental stability, canalization, and phenotypic plasticity play important roles in phenotypic expression, and they should not be necessarily independent, particularly in the stressful contexts (Hoffmann & Parsons, 1991). The associations between the three mechanisms have been paid increasingly greater attention in recent twenty years (Debat & David, 2001), although they have been considered separately for a long time. Actually, how these mechanisms interact to generate phenotypic variation has become a key focus of “eco‐evo‐devo” (Pfennig, 2016).

It has been disputed whether the underlying mechanisms for developmental stability and canalization are independent/different (Debat et al., 2000), or overlapping/related (Debat et al., 2009; Lazić et al., 2016), and whether plasticity and developmental stability have correspondence (Willmore et al., 2005; Woods et al., 1999) or not (Debat et al., 2000; Milton et al., 2003). The controversies suggest these relationships are complex and depend on other factors such as specific traits, environmental contexts, and growth stages (Woods et al., 1999), and direct evidence is lacking. In addition, another related concept that has recently been given more attention is “intraindividual variability,” which is reported to play important roles in determining the ability of plants to deal with environmental changes (March‐Salas et al., 2021) and improving distribution of species and population stability and persistence (Herrera et al., 2015). Environmental effects can alter intraindividual variability without affecting plant average performance (Gonzalez‐Jimena & Fitze, 2012) or intrapopulation variability (Herrera et al., 2015). However, investigation is scarce on intraindividual variation and its relationships with other variables. If developmental stability and variability, canalization, and plasticity all play important roles in species survival and adaptation (Kawano, 2020), there should be some associations between them (Debat & David, 2001). Unfortunately, most relevant studies, which mainly focus on animals, have attempted to speculate their possible connections by comparative studies (Debat et al., 2000, 2009; Woods et al., 1999), with rare direct evidence on concrete correlations among developmental stability and variability, canalization, and plasticity (but see Tonsor et al., 2013; Tucić et al., 2018). Furthermore, plants should be more ideal materials than animals for addressing associations between these mechanisms, since they are sessile and can only rely on regulatory mechanisms to cope with environmental variabilities (Sultan, 2000). Besides, the architectural characteristics should also be an advantage of plants over animals for dynamic and correlative analyses on phenotypic variations (de Kroon et al., 2005), since plants (modular organisms) have repetitive modules continuously produced over the entire lifetime.

For plant species, population density is one of the major natural biotic environmental factors that have profound effects on their survival, growth, and reproduction (Wang, Li, et al., 2017; Zhou et al., 2005). Increased density can lead to variations in different abiotic and biotic factors, inducing complex plasticity in traits (Wang, Li, et al., 2017; Wang et al., 2021). In response to increased density or shade, plants can alter leaf traits such as leaf size, petiole length, and leaf number, producing substantial plasticity in leaves, which vary with soil conditions and plant growth stages (Balaguer et al., 2001; Wang, Li, et al., 2017). The plasticity to density in leaf size may correlate with its developmental stability (Valladares et al., 2002) and canalization (Balaguer et al., 2001; Kawecki, 2000; Lamy et al., 2014). Increased density can trigger the covariations of these processes, leading to significant correlations among developmental stability, canalization, and plasticity (Wang & Zhou, 2021a). Correlations may also depend on the strength of environmental selections (Kawecki, 2000; Wang & Zhou, 2021a), due to effects of other factors such as abiotic conditions and plant growth stage.

Since plant performance in natural conditions differs remarkably from that in laboratory (Poorter et al., 2012), we conducted a field experiment to investigate the relationships among leaf developmental stability, variability, canalization, and plasticity in plants. Plants of an annual herbaceous species, *Abutilon theophrasti*, were subjected to three different population densities in fertile *versus* infertile soil conditions in the field. The leaf width (leaf size) and the left and right width were measured for all leaves on the main stem of each individual plant at three growth stages, in order to calculate leaf fluctuating asymmetry (FA), intra‐ and interindividual coefficient of variations (CV_intra_ and CV_inter_), and plasticity (PI) of leaf size and analyze their correlations. We aimed to answer the following questions: (1) Are there any correlations among leaf FA, CV_intra_, CV_inter_, and PI in plants? And (2) do these correlations vary with different densities or soil conditions at each of or across all growth stages?

## 2 MATERIALS AND METHODS

### 2.1 Study species

*Abutilon theophrasti* Medicus (Malvaceae) is an annual weedy species (Figure A1). It grows rapidly to a height of up to 1–1.5 m (Gleason & Cronquist, 1991), reaching reproductive maturity within 90 days, and completing its life cycles in about five months (McConnaughay & Coleman, 1999). It colonizes relatively nutrient‐rich habitats, being ubiquitous in open fields, on roadsides, and in gardens, with substantial plasticity in allocation, morphology, and architecture in response to varying environmental factors (McConnaughay & Bazzaz, 1992).

### 2.2 Experimental design

The experiment was conducted between June and August in 2007 at the Pasture Ecological Research Station of Northeast Normal University, Changling, Jilin Province, China (123°44 E, 44°40 N). We collected seeds of *A*. *theophrasti* from the local wild populations near the research station in late August 2006 and dry‐stored them at −4°C. The experiment applied a split‐plot design, with soil conditions as main factor, and density and block as subfactors. Two main plots were assigned as infertile and fertile soil conditions, and each plot was divided into nine 2 × 3 m subplots and randomly distributed with three density treatments and three blocks. Seeds were sown on June 7, 2007, with distances of 30, 20, and 10 cm apart, to reach the target plant densities of 12.8, 27.5, and 108.5 plants·m^−2^, representing relatively low‐, medium‐, and high‐density treatments, respectively. Most seeds emerged four to five days after sowing. Seedlings were then thinned to the target densities when a majority of individuals reached four‐leaf stage. Plots were hand‐weeded when necessary and watered regularly ad libitum to prevent drought.

We set up the plot of infertile soil conditions on the original soil of experimental field at the station (aeolian sandy soil in low nutrient availability of organic C 3.1 mg·kg^−1^, available N 21.0 mg·kg^−1^, and available P 1.1 mg·kg^−1^), due to frequent cultivations for many years. The treatment of fertile soil conditions was set up by covering the other plot with 5‐ to 10‐cm virgin soil (meadow soil, pH = 8.2, with main nutrients of organic C 18.7 mg·kg^−1^, available N 47.5 mg·kg^−1^, and available P 4.0 mg·kg^−1^), transported from the nearby meadow with no cultivation history (for details on soil conditions, see Wang & Zhou, 2021a).

### 2.3 Data collection and analysis

Plants were sampled at days 30, 50, and 70 of growth, representing stages of early vegetative growth, late vegetative or early reproductive growth, and middle–late reproductive growth. At each stage, five to six individuals were randomly chosen from each plot, making a maximum total of 6 replicates × 3 blocks × 3 densities × 2 soils × 3 stages = 324 individuals sampled. Samples from different treatments and blocks were mixed together and measured in a random sequence. For each individual plant, we measured all the leaves on the main stem immediately after sampling when they were fresh. For each leaf, we used digital calipers to measure the width of right and left halves (from the midrib to the leaf margin) at the widest point of a leaf, perpendicular to the midrib (Wilsey et al., 1998). The width of each of the sides was measured twice successively and immediately after each other. Leaf size (LS) was calculated as the average width of right and left sides (Palmer & Strobeck, 1986; Wilsey et al., 1998). To calculate the fluctuating asymmetry (FA) in leaf width, various conventional indices (FA_1_–FA_8_ and FA_10_) were compared to identify the ones with the highest explanatory powers for our study design (Table A1). Different indices showed similar trends in response to various factors (Tables A2 and A3; Figure A2). We finally adopted FA_1_, FA_2_ (with and without effects of leaf size, respectively), and FA_10_ (the only index with measurement error variance partitioned out of the total between‐sides variance) in analyses, with the formula as (Palmer, 1994; Palmer & Strobeck, 2003):

(1) $${\text{FA}}_{1}=\sum \left|R‐L\right|/n$$

(2) $${\text{FA}}_{2}=\sum \left[\left|R‐L\right|/\text{LS}\right]/n$$

(3) $${\text{FA}}_{10}=0.798\times \surd \left({\text{MS}}_{\mathrm{si}}‐{\text{MS}}_{m}\right)/M$$

where *R* and *L* were the widths of right and left sides of a leaf, *n* was the total number of leaves, and LS was leaf size and calculated by (*R*+*L*)/*2*, MS*_si_* was the mean squares of side × individual interaction, MS*_m_* was the mean squares of measurement error, and *M* was the number of replicate measurements per side, from a side × individual ANOVA on untransformed replicate measurements of *R* and *L*.

We measured skew (*γ*_1_) and kurtosis (*γ*_2_) to evaluate whether the leaf asymmetry deviated from normality. To test the presence of directional asymmetry, we used two methods: (1) testing (*R*–*L*) against 0 with one‐sample *t* test (the hypothesis *H*_0_:*γ*_1_ = 0); and (2) testing whether the difference between sides (mean squares for side effect [MS*_s_*]) is greater than nondirectional asymmetry (mean squares for side × individual interaction [MS*_si_*]) with factorial ANOVA (Palmer, 1994; Wilsey et al., 1998). To detect the presence of antisymmetry, kurtosis (*γ*_2_) was tested with a *t* test of the null hypothesis H_0_:*γ*_2_ = 0, where a significant negative*γ_2_* indicates possible antisymmetry (Cowart & Graham, 1999; Palmer, 1994). The individuals of three cases (density and stage combination) showed right‐dominant directional asymmetry, and two cases showed left‐dominant directional asymmetry (Table A4). Individuals of three cases also showed a greater mean difference between sides than between‐sides variation (Table A5), indicating directional asymmetry. Two sets of samples showed leptokurtosis, indicating antiasymmetry (Table A4). To determine the size dependence of leaf asymmetry, we regressed |*R*–*L*| on LS for all the leaves of individuals at each density and stage, and found leaf asymmetry in most cases was size‐dependent. We also evaluated whether the between‐sides variation (MS*_si_*) is significantly larger than the measurement error (MS*_m_*) in factorial ANOVA (Palmer, 1994). The MS*_m_* values for all treatments were lower than MS*_si_* values (Table A5).

Canalization in leaf size was evaluated by coefficient of variation (CV, the standard deviation divided by the mean value of a trait) among individuals (CV_inter_). CV among leaves within each individual (CV_intra_) was calculated as developmental variability or intraindividual variability (Woods et al., 1999).

The level of plasticity in leaf size, or relative plasticity, was calculated by simplified Relative Distance Plasticity Index (RDPI*_s_*; Valladares et al., 2006) and abbreviated as PI_rel_, with the degree of plasticity or absolute plasticity in leaf size abbreviated as PI_abs_. PI_rel_ and PI_abs_ were calculated with the formulas as:

(4) $${\text{PI}}_{\text{rel}}=\left(X‐Y\right)/\left(X+Y\right)$$

(5) $${\text{PI}}_{\text{abs}}=\left|X‐Y\right|/\left(X+Y\right)$$

where *X* is the adjusted mean leaf size at high or medium density, and *Y* is the adjusted mean leaf size at low density. We calculated both relative and absolute plasticity in response to high vs. low density (PI_rel‐HL_ and PI_abs‐HL_) and in response to medium vs. low density (PI_rel‐ML_ and PI_abs‐ML_). Adjusted mean values of leaf size were produced from one‐way ANCOVA on original mean leaf size, with density as effect and plant size (total mass) as a covariate.

All variables were used in statistics, and the data of measured leaf width were log‐transformed to minimize variance heterogeneity before any analysis. All analyses were conducted using SAS statistical software (SAS Institute 9.0 Incorporation 2002). Three‐way ANOVA was performed for overall effects of growth stage, soil conditions, population density, and their interactions on all variables. Then, we used one‐way ANOVA for effects of density on all variables in each soil condition at each stage or across all soils and stages. Multiple comparisons used the least significant difference method (LSD) in general linear model (GLM) program. For each of and across all treatments, correlations among leaf size, leaf FA_2_, CV_intra_, CV_inter_, and PI_rel_ were analyzed with PROC CORR, producing Pearson's correlation coefficients (PCCs) for all correlations and partial Pearson's correlation coefficients (PPCCs) for correlations among leaf FA_2_, CV_intra_, CV_inter_, and PI_rel_, with leaf size in control in partial correlation analyses.

## 3 RESULTS

### 3.1 Responses of different variables to density

**FIGURE 1:**
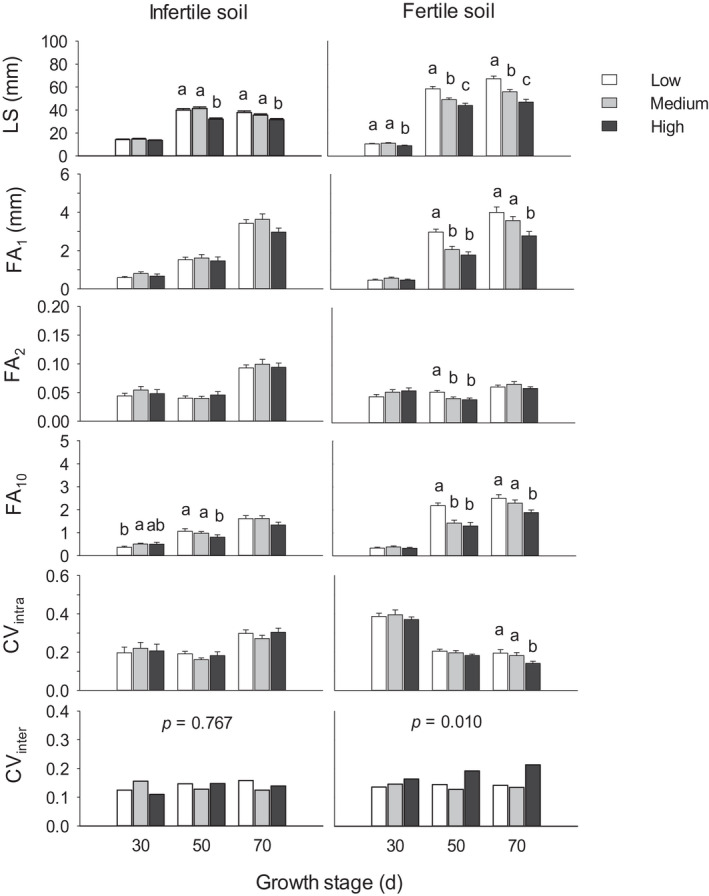
Mean values (±SE) of leaf size (LS), fluctuating asymmetry (FA_1_, FA_2_ and FA_10_) of leaf width, and intraindividual and interindividual coefficient of variation (CV_intra_ and CV_inter_) in response to density, for plants in infertile (left) and fertile (right) soil conditions at days 30, 50, and 70 of plant growth. Different letters denote significant differences between density treatments within each of the soil conditions and growth stage (LSD, *p* < .05); *p*‐values (from LSD) indicate differences between densities across all stages

**FIGURE 2:**
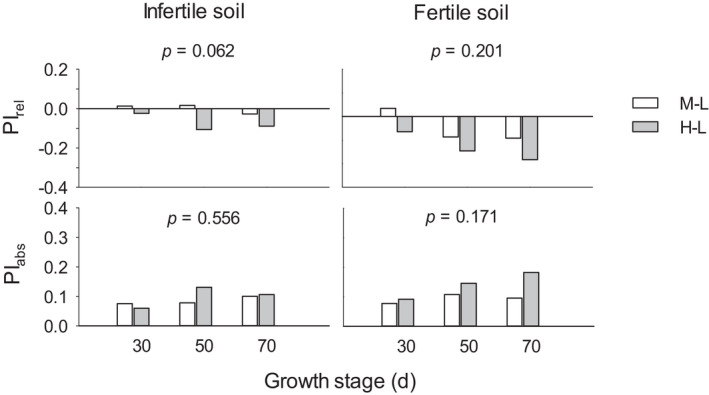
Relative plasticity (PI_rel_) and absolute plasticity (PI_abs_) of leaf size in response to medium vs. low density (M–L) and high vs. low density (H–L) in infertile (left) and fertile (right) soil conditions at days 30, 50, and 70 of plant growth. The *p*‐values (from LSD) indicate differences between densities across all stages in each of the soil conditions

Soil condition, growth stage, and population density, and their interactions had significant effects on leaf size and fluctuating asymmetry (FA_1_ and FA_10_); effects of soil conditions, growth stage, and their interaction were significant for FA_2_ and intraindividual variation (CV_intra_); effects of soil conditions, population density, and their interaction were significant for interindividual variation (CV_inter_); and little effect was found for plasticity (PI_rel_ and PI_abs_; Table 1). In fertile soil, leaf size decreased with higher densities at all stages (LSD, *p* < .05); in infertile soil conditions, leaf size was smaller at high density than at low and medium densities at stages of day 50 and 70 (*p* < .05; Figure 1). In fertile soil, high density also decreased FA_1_ and FA_10_ at days 50 and 70 (*p* < .01), decreased FA_2_ at Day 50 and CV_intra_ at Day 70 (*p* < .05), and increased CV_inter_ significantly across days 50 and 70 (*p* < .01). In infertile soil, high density decreased FA_10_ at Day 50 (*p* = .040), whereas medium density increased it at Day 30 (*p* = .045), compared with that at low density. Relative plasticity (PI_rel_, the level of plasticity) in response to high vs. low density (PI_rel‐HL_) was slightly lower than that in response to medium vs. low density (PI_rel‐ML_) across days 50 and 70 in fertile (*p* = .057) and infertile (*p* = .059) soil conditions (Figure 2).

**TABLE 1:** *F*‐values for three‐way ANOVA on fluctuating asymmetry (FA_1_, FA_2_, and FA_10_), coefficients of variation (CV_intra_ and CV_inter_), and phenotypic plasticity (PI_rel_ [relative plasticity, or the level of plasticity] and PI_abs_ [absolute plasticity, or the degree of plasticity]) with soil conditions (SC), growth stage (GS), population density (PD), and their interactions as effects

Source of variation	df	Log_10_ (LS)	FA_1_	FA_2_	FA_10_	CV_intra_	CV_inter_	PI_rel_	PI_abs_
SC	1	54.90***	5.73*	14.82***	66.82***	5.05*	13.84*	3.20	4.45
GS	2	2320.60***	339.73***	88.84***	254.44***	58.35***	2.10	3.28	5.87
PD	2	59.68***	12.35***	0.38	14.51**	1.01	8.80*	4.65	3.03
SC × GS	2	175.36***	9.66***	25.47***	23.21***	90.76***	0.24	0.36	0.53
SC × PD	2	5.00**	6.18**	0.54	4.97**	2.16	14.97*	0.49	1.00
GS × PD	4	3.76**	3.95**	1.51	5.33***	0.55	5.18	0.52	1.24

Note

*p < .10, **p < .05, and ***p < .01.

### 3.2 Correlations among different variables

PI_rel_ negatively correlated with leaf size at all densities and in infertile soil, with Pearson's correlation coefficients (PCCs) ranging from −0.843 to −0.952, though correlations among FA_2_, CV_intra_, CV_inter_, and PI_rel_ were nonsignificant in most cases (Tables 2 and 3). There were more negative correlations between PI_rel_ and FA in Pearson's correlation analyses, but with analyses of partial Pearson's correlation (leaf size in control), we found only one case of positive correlation between PI_rel_ and FA_10_ (with PPCC of 0.731) in infertile soil (Table 2). PI_rel_ also negatively correlated with CV_inter_ at high density (PPCC −0.950) and in fertile soil (PPCC −0.720) across all the other treatments. We did not find significant correlations between FA_2_ and CV_intra_, but leaf size had significantly negative correlations with FA_2_ or CV_intra_ in a few cases (Table 3).

**TABLE 2:** Pearson's correlation coefficients (PCCs) and partial Pearson's correlation coefficients (PPCCs) for correlations of mean leaf size, leaf fluctuating asymmetry (FA_2_ and FA_10_), and intraindividual coefficient of variation (CV_intra_) with interindividual coefficient of variation (CV_inter_) and relative plasticity in response to medium vs. low density (PI_rel‐ML_) and in response to high vs. low density (PI_rel‐HL_) across all stages and soils at low, medium, and high densities

Density/soil	Trait	Coefficient	LS	FA_2_	FA_10_	CV_intra_	CV_inter_
Low	CV_inter_	PCC	0.503	0.718	0.558	0.112	
		PPCC		0.686	0.350	0.551	
	PI_rel‐HL_	PCC	−0.952**	−0.214	−0.926**	0.365	−0.478
		PPCC		0.258	0.024	−0.664	0.003
	PI_rel‐ML_	PCC	−0.912*	−0.392	−0.955**	0.461	−0.342
		PPCC		−0.295	−0.721	−0.149	0.329
Medium	CV_inter_	PCC	−0.751	−0.257	−0.640	0.366	
		PPCC		−0.352	0.091	−0.370	
	PI_rel‐ML_	PCC	−0.843*	−0.153	−0.868*	0.499	0.488
		PPCC		−0.232	−0.484	−0.277	−0.409
High	CV_inter_	PCC	0.704	−0.153	0.681	−0.327	
		PPCC		−0.229	0.125	0.318	
	PI_rel‐HL_	PCC	−0.920**	0.051	−0.865*	0.559	−0.912*
		PPCC		0.165	−0.129	−0.288	−0.950*
Infertile	CV_inter_	PCC	0.313	0.159	0.272	0.247	
		PPCC		0.082	0.047	0.220	
	PI_rel‐HL_	PCC	−0.904***	0.020	−0.510	0.116	−0.389
		PPCC		0.630	0.731*	0.543	−0.262
	PI_rel‐ML_	PCC	−0.090	−0.224	−0.162	−0.180	−0.096
		PPCC		−0.209	−0.147	−0.171	−0.072
Fertile	CV_inter_	PCC	−0.008	−0.015	0.008	−0.319	
		PPCC		−0.013	0.071	−0.783*	
	PI_rel‐HL_	PCC	−0.626	−0.342	−0.661*	0.626	−0.556
		PPCC		−0.200	−0.292	0.174	−0.720*
	PI_rel‐ML_	PCC	−0.666	−0.373	−0.665*	0.719*	−0.225
		PPCC		−0.235	−0.094	0.365	−0.309

Note

*p < .05, **p < .01, and ***p < .001.

**TABLE 3:** Pearson's correlation coefficients (PCCs) and partial Pearson's correlation coefficients (PPCCs) for correlations among mean leaf size (LS), leaf fluctuating asymmetry (FA_2_), and intraindividual coefficient of variation (CV_intra_) for plants at low, medium, and high densities under two soil conditions at growth stages of days 30, 50, and 70

Soil	Density	Trait	Stage (day)	30		50		70	
			Coefficient	LS	CV_intra_	LS	CV_intra_	LS	CV_intra_
Infertile	Low	CV_intra_	PCC	−0.347		−0.313		0.038	
		FA_2_	PCC	−0.493	−0.284	−0.525*	0.430	−0.23	0.041
			PPCC		−0.557		0.329		0.053
	Medium	CV_intra_	PCC	−0.536		−0.254		−0.435	
		FA_2_	PCC	0.231	−0.333	0.036	0.275	−0.119	0.154
			PPCC		−0.255		0.294		0.114
	High	CV_intra_	PCC	0.182		−0.122		0.123	
		FA_2_	PCC	−0.045	−0.159	0.176	0.120	−0.173	−0.040
			PPCC		−0.153		0.145		−0.019
Fertile	Low	CV_intra_	PCC	0.047		0.214		−0.231	
		FA_2_	PCC	−0.083	0.192	−0.333	0.099	0.014	0.370
			PPCC		0.189		0.185		0.383
	Medium	CV_intra_	PCC	0.463		0.234		−0.610**	
		FA_2_	PCC	−0.392	−0.472	0.331	0.309	−0.585*	0.282
			PPCC		−0.356		0.252		−0.148
	High	CV_intra_	PCC	0.341		0.177		−0.402	
		FA_2_	PCC	−0.136	0.397	0.106	0.159	0.131	0.196
			PPCC		0.476		0.144		0.274

Note

*p < .05, **p < .01, and ***p < .001.

## 4 DISCUSSION

### 4.1 Responses of variables to density

#### 4.1.1 Developmental stability

It is generally regarded that environmental stresses can induce higher levels of fluctuating asymmetry (FA) in traits, indicating higher developmental instability (Hagen et al., 2008; Møller, 1998). However, our results showed leaf FA of *Abutilon theophrasti* was reduced by increased density, consistent with other results (Kruuk et al., 2003). It may be because FA is an unreliable indicator of environmental stresses (Abeli et al., 2016; Palmer & Strobeck, 2003), and the relationships between developmental stability and environmental conditions are often complicated and not simply in correspondence (Bonduriansky, 2009; Woods et al., 1999). Some researchers argue that favorable environments may allow faster growth of plants or modules, prompting higher developmental instability and FA levels (Martel et al., 1999; Morris et al., 2012). Increased FA has been found in higher nutrient availability (Milligan et al., 2008), less polluted soil (Velickovic & Perisic, 2006), or water supplementation (Fair & Breshears, 2005). Therefore, developmental instability or higher FA may not be harmful, but simply reflect the state of fast growth in modules or organisms (Morris et al., 2012), or that environments are relatively favorable. Since the fast‐growing state of organisms also indicates immature stage, these organisms should be less suitable for mating or digestion than the more mature or stable ones (Cornelissen & Stiling, 2005). It may have explained why animals prefer to choose the spouses or plants with fitness‐related traits with lower levels of FA (Møller & Eriksson, 1994; Møller & Thornhill, 1998).

In this sense, lower FA reflected adverse effects of increased density and the state of slow growth of *A*. *theophrasti* at higher densities. Nevertheless, FA did not decrease with higher densities in all cases. It implied whether leaf FA increase or decrease with stress depended on the strength of stress; moderate stress (e.g., weak aboveground competition in infertile soil conditions) will be more likely to induce higher FA, while intense stress (e.g., strong aboveground competition in fertile soil) tends to decrease it. Sometimes, asymmetry also increases with leaf size because larger leaves require more resources and grow faster (Møller & Eriksson, 1994). However, we found negative correlations between leaf FA and leaf size at low (Day 50) or medium (Day 70) density, probably because smaller leaves grew faster than larger ones in more benign environment.

#### 4.1.2 Developmental variability

Similar to FA, intraindividual variation (CV_intra_) of leaf size also decreased with higher densities at Day 70 in fertile soil. Plants of *A*. *theophrasti* tend to have smaller leaves with higher layers (vertical positions along the main stem) at low density, but had canalized or greater leaves in upper layers and smaller leaves in low or middle layers at higher densities (Wang & Zhou, 2022). Plants grown with neighbors will enhance leaf size and petiole length in upper layers to locate foliage higher above other plants to maximize light acquirement (Van de Peer et al., 2017; Yang et al., 2019), while reducing them in lower layers to save energy (Wang & Zhou, 2022). Consequently, variations among different layers in leaf size decreased with higher densities. Significant responses of plant architecture and intraindividual variations to increased density suggested intense competition among plants at Day 70 in fertile soil (Wang & Zhou, 2021b).

#### 4.1.3 Canalization

Both FA and CV_intra_ had more pronounced decreases with increased density, due to stronger aboveground competition, in fertile vs. infertile soil conditions (Wang, Li, et al., 2017). By contrast, the interindividual variation (CV_inter_) in leaf size increased with higher densities in fertile soil, probably because there were more small plants in dense populations, leading to greater variation in plant size and leaf size than sparse populations. Fertile soil should have aggravated aboveground competition among plants of dense populations, leading to a more remarkable increase in CV_inter_ of leaf size than in infertile soil (Wang & Zhou, 2021a).

### 4.2 Correlations among variables

All the mechanisms of developmental stability (FA), variability (CV_intra_), canalization (CV_inter_), and phenotypic plasticity (PI_rel_) have a genetic basis (Leamy & Klingenberg, 2005; Pigliucci, 2005; Violle et al., 2012; Wagner, 1996). They could be independent components on their own and potentially part of important evolutionary processes (Bradshaw, 1965; Herrera et al., 2015). Meanwhile, they are also under selection (Kawecki, 2000; March‐Salas et al., 2021; Møller & Swaddle, 1997; Pigliucci et al., 2006). The presence of correlative relationships among them might simply reflect their similar trends in response to environmental gradients as a coincidence, and they do not have any actual correlations, or otherwise, they can have some common mechanisms and explain each other (Del Giudice et al., 2018; McDonald et al., 2018). In the former case, correlations among them should not display any pattern or rule, but just occur randomly along environmental gradients (Debat et al., 2000; Milton et al., 2003). Our results, however, showed the contrary fact, thereby inclining to support the latter case. We found significant correlations among FA, CV_intra_, CV_inter_, and PI_rel_ more frequently at high density or in fertile soil, where aboveground competition among plants was stronger than otherwise cases, though most correlations were nonsignificant. The results suggested that both negative and positive correlations may occur among developmental stability, variability, canalization, and phenotypic plasticity; the overall results can be either positive, negative, or nonsignificant, depending on specific circumstances. This may explain the inconsistent hypotheses on this issue in different studies.

#### 4.2.1 Correlations between developmental stability and canalization

Developmental stability and canalization are argued to evolve independently (Debat et al., 2000) or have overlapping mechanisms (Debat et al., 2009; Lazić et al., 2016). We found nonsignificant correlations of FA with CV_intra_ or CV_inter_, but our other results showed more positive than negative correlations between FA and CV_inter_ for leaf size, petiole length, and angle at lower densities than at high density (unpublished data), consistent with other results (Nagamitsu et al., 2004). These results suggested the complexity of the relationships among different mechanisms, and positive correlations among developmental instability, variability, and decreased canalization are more likely to occur in relatively less stressful environments.

#### 4.2.2 Correlations between developmental stability and plasticity

Relevant studies either suggest correspondence between plasticity and developmental stability (Willmore et al., 2005; Woods et al., 1999) or the contrary (Debat et al., 2000; Milton et al., 2003), but direct evidence is rare. The results on seedlings of two oak species from the Mediterranean basin have suggested a positive correlation between phenotypic plasticity and developmental instability (Valladares et al., 2002). Our results showed one case of positive correlation between FA and PI_rel_ in infertile soil, and more cases of such positive correlations can be found in other studies (Tonsor et al., 2013; Tucić et al., 2018). These results suggested the relationship between developmental instability and plasticity is also complex and depends on specific circumstances (Wang & Zhou, 2021a). Developmental instability can increase to facilitate plant adaptive responses in less stressful environment, for instance, when aboveground competition was not intense in infertile soil, leading to more positive correlations between developmental instability and plasticity. Alternatively, it can also decrease to stabilize performance in more severe stress, when negative correlations between developmental instability and plasticity increased, counteracting positive correlations, leading to nonsignificant or negative overall results of correlations.

#### 4.2.3 Correlations between canalization and plasticity

Genetic canalization is said to constrain phenotypic response (Kawecki, 2000; Lamy et al., 2014), and the greater phenotypic plasticity due to ecotypic divergence can promote genetic variation (Balaguer et al., 2001). It implies a negative correlation between plasticity and canalization, yet with rare direct evidence. Our results showed negative correlations between CV_inter_ and PI_rel_ at high density or in fertile soil, suggesting that higher interindividual variation is more likely to coincide with lower plasticity (decrease in leaf size) when aboveground competition was more intense. We also found more positive than negative correlations between CV_inter_ and PI_rel_ across different layers in leaf traits at lower densities vs. high density in another study (unpublished data). These demonstrated that decreased canalization may be disadvantageous or advantageous, leading to either negative or positive correlations between canalization and plasticity, depending on specific environments (Kawecki, 2000; Wang & Zhou, 2021a). Correlations between decreased canalization and plasticity should more likely be positive in less stressful conditions, while tend to become less positive or more negative under more stressful conditions (Wang & Zhou, 2021a).

#### 4.2.4 Correlations between developmental variability and plasticity

The negative correlations between CV_intra_ and CV_inter_ and between CV_inter_ and PI_rel_ implied positive correlations between CV_intra_ and PI_rel_, at high density or in fertile soil. However, we found nonsignificant correlations between CV_intra_ and PI_rel_. Since increased intraindividual variation can either be beneficial for plastic response (March‐Salas et al., 2021) or reflect adverse environmental effects, both positive and negative correlations can occur between intraindividual variation and plasticity, with overall results being nonsignificant.

## 5 CONCLUSIONS

Our results showed the decrease in FA and CV_intra_ and increase in CV_inter_ in response to increased density were more pronounced in fertile vs. infertile soil, probably due to intense aboveground competition in abundance of resources. Results suggested responses of these variables to density largely depended on the strength of aboveground competition among plants, which varied with soil conditions. Moderate aboveground competition should be more likely to induce higher developmental instability, variability, and canalization, whereas intense aboveground competition tends to reduce them. Furthermore, occasional positive or negative correlations among different variables and nonsignificant correlations in other cases suggested relationships among developmental instability, intra‐ and interindividual variability, and plasticity are complex, the overall results depending on the strength of environmental selections. In less stressful conditions, increased developmental instability, and intra‐ and interindividual variability are beneficial and can facilitate adaptive responses (less decrease or more increase) in traits; thus, they are more likely to have positive correlations with plasticity. In more stressful conditions, however, greater developmental instability, and intra‐ and interindividual variability are less advantageous than otherwise for stabilizing performance of phenotype; thus, they may have more negative correlations with plasticity, counteracting positive correlations, leading to nonsignificant or negative overall results. This may have explained to a large extent the inconsistent conclusions from different relevant studies. Future studies examining the dynamic patterns of responses to developmental instability, intra‐ and interindividual variability and plasticity, and their correlations to various environments can provide more direct evidence in detail for our hypotheses.

In the complicated natural world that is ever‐changing, to change or not change may always be a paradox that an organism is confronted with. Any pattern of phenotypic variations, with or without genetic basis, may not necessarily be absolutely advantageous or disadvantageous. The adaptive significance of these variations should be interpreted depending on specific circumstances. For example, in some cases, phenotypic variations such as decreased performance in biomass appearing to be nonadaptive currently or in a short term in one perspective can have adaptive significance later or from a different perspective (Ghalambor et al., 2015; Wang, Callaway, et al., 2017). Organisms are able to deal with environmental changes relying on regulating mechanisms for variability or invariability and their interactions, keeping the developmental system flexible (both relatively stable and appropriately plasticity). Overall, the world of life is always dynamically stable and elastic, because of its vitality.

## CONFLICT OF INTEREST

The authors have no conflict of interest to declare.

## AUTHOR CONTRIBUTIONS

**Shu Wang:** Conceptualization (lead); Data curation (lead); Formal analysis (lead); Funding acquisition (lead); Investigation (lead); Methodology (lead); Project administration (lead); Resources (lead); Software (lead); Supervision (lead); Validation (lead); Visualization (lead); Writing – original draft (lead); Writing – review & editing (lead). **Dao‐Wei Zhou:** Conceptualization (supporting); Funding acquisition (supporting); Methodology (supporting).

## Supporting information

Appendix S1

## Data Availability

Data are available via the Dryad Digital Repository: https://doi.org/10.5061/dryad.612jm643m.
