# Supplementary material for: Associations between leaf developmental stability, variability, canalization, and phenotypic plasticity in Abutilon theophrasti
Source: Ecol Evol. 2022 Apr 17;12(4):e8845. doi: 10.1002/ece3.8845 (PMC9013853; doi:10.1002/ece3.8845)
Supplement: Supplementary file 1 — Appendix S1 [file ECE3-12-e8845-s001.docx]

**Appendix 1**

**Table A1** All the formulas for FA indexes (Palmer and Strobeck 1986, 1994, 2003) used in this study. *R* and *L* were the widths of right and left sides of a leaf, *n* was the total number of leaves, and *LS* (leaf size) was calculated by (*R*+*L*)/*2*, *MS_sj_* was the mean squares of side × individual interaction, *MS_m_* was the mean squares of measurement error, *M* was the number of replicate measurements per side, from a side × individual ANOVA on untransformed replicate measurements of *R* and *L*.

| Index | Formula |
| --- | --- |
| FA_1_ | *mean*│*R* - *L*│ |
| FA_2_ | *mean* (│*R* - *L*│/ *LS*) |
| FA_3_ | *mean*│*R* - *L*│/ *mean LS* |
| FA_4_ | *0.798* ×√var (*R* - *L*) |
| FA_5_ | *0.798* × [ ∑(*R* - *L*)^2^ / *n*] |
| FA_6_ | *0.798*×√var [(*R* - *L*) / *LS*] |
| FA_7_ | *0.798*×√var (*R* - *L*) / *mean* *LS* |
| FA_8_ | *mean*│ln(*R*/*L*)│ |
| FA_10_ | *0.798* × √ (*MS_sj_* - *MS_m_*) / *M* |

**Table A2** *F*-values for three-way ANOVA on mean values of leaf size (LS) and FA indexes with growth stage (GS), soil conditions (SC), population density (PD) and individual (IN) nested in density as effects. * *p* < 0.05, ** *p* < 0.01, *** *p* < 0.001.

| Source of variation | Df. | LS | FA_1_ | FA_2_ | FA_4_ | FA_5_ | FA_6_ | FA_8_ |
| --- | --- | --- | --- | --- | --- | --- | --- | --- |
| SC | 1 | 380.55*** | 34.45*** | 7.31** | 3.99* | 29.88*** | 4.78* | 3.90* |
| GS | 2 | 3685.71*** | 267.20*** | 86.07*** | 0.76 | 130.57*** | 3.72* | 5.52** |
| PD | 2 | 104.13*** | 16.33**** | 0.25 | 2.22 | 14.02*** | 2.90 | 0.82 |
| IN(PD) | 53 | 3.08*** | 1.35* | 0.75 | 2.12*** | 1.15 | 2.05*** | 2.01*** |
| SC*GS | 2 | 250.61*** | 8.74*** | 32.89*** | 4.03* | 3.08* | 5.04** | 4.01* |
| SC*PD | 2 | 9.16*** | 5.50** | 0.58 | 5.42** | 6.01** | 5.79** | 3.80* |
| GS* PD | 4 | 4.06** | 2.75* | 1.77 | 0.69 | 3.69** | 0.37 | 0.88 |
| GS*SC*PD | 4 | 3.10* | 0.83 | 1.23 | 2.67* | 0.71 | 3.08* | 3.08* |

Degree of freedom for the error terms were 1854.

**Table A3** *F*-values for one-way ANCOVA on all FA indexes with population density (PD) as effect, and leaf size (LS) as covariate, for plants in infertile and fertile soil conditions and Day 30, 50 and 70 growth stages. * *p* < 0.05, ** *p* < 0.01, *** *p* < 0.001.

| Source of variation | Day 30 |  |  | Day 50 |  |  | Day 70 |  |  | All stages |  |  |
| --- | --- | --- | --- | --- | --- | --- | --- | --- | --- | --- | --- | --- |
|  | LS  (Df=1) | PD  (Df=2) | LS*PD  (Df=2) | LS  (Df=1) | PD  (Df=2) | LS*PD  (Df=2) | LS  (Df=1) | PD  (Df=2) | LS*PD  (Df=2) | LS  (Df=1) | PD  (Df=2) | LS*PD  (Df=2) |
| Infertile soil | |  |  |  |  |  |  |  |  |  |  |  |
| FA_1_ | 0.46 | 0.27 | 0.33 | 2.27 | 1.64 | 1.68 | 1.68 | 0.045 | 0.064 | 56.52*** | 0.088 | 0.071 |
| FA_2_ | 1.00 | 0.36 | 0.40 | 0.072 | 2.06 | 2.09 | 1.33 | 0.001 | 0.003 | 3.71 | 0.52 | 0.53 |
| FA_3_ |  |  |  |  |  |  |  |  |  | 0.34 | 0.03 | 0.023 |
| FA_4_ | 0.10 | 0.19 | 0.22 | 3.91 | 3.76* | 3.76* | 0.42 | 0.26 | 0.32 | 53.97*** | 0.039 | 0.049 |
| FA_5_ | 0.25 | 0.13 | 0.16 | 3.57 | 2.47 | 2.51 | 0.32 | 0.18 | 0.22 | 58.08*** | 0.077 | 0.059 |
| FA_6_ | 1.67 | 0.37 | 0.40 | 0.13 | 5.88** | 5.86** | 4.44* | 0.34 | 0.41 | 0.19 | 0.109 | 0.14 |
| FA_7_ |  |  |  |  |  |  |  |  |  | 0.021 | 0.102 | 0.080 |
| FA_8_ | 0.035 | 1.42 | 1.35 | 0.028 | 1.29 | 1.40 | 6.81* | 1.85 | 1.89 | 0.05 | 1.18 | 1.44 |
| FA_10_ | 0.24 | 0.11 | 0.12 | 0.31 | 1.58 | 1.49 | 0.42 | 0.25 | 0.31 | 48.31*** | 0.36 | 0.41 |
| Fertile soil | |  |  |  |  |  |  |  |  |  |  |  |
| FA_1_ | 2.38 | 0.63 | 0.56 | 13.03** | 1.05 | 0.93 | 10.41*** | 2.47 | 2.43 | 309.90*** | 2.07 | 3.62* |
| FA_2_ | 1.30 | 0.22 | 0.17 | 0.03 | 2.49 | 2.27 | 2.89 | 4.13* | 3.93* | 0.67 | 0.11 | 2.59 |
| FA_3_ |  |  |  |  |  |  |  |  |  | 0.12 | 0.17 | 0.18 |
| FA_4_ | 0.42 | 1.19 | 1.11 | 12.33** | 0.45 | 0.36 | 5.86* | 3.95* | 3.91* | 337.32*** | 0.16 | 3.30* |
| FA_5_ | 1.70 | 0.53 | 0.45 | 10.08** | 0.38 | 0.30 | 6.60* | 3.52* | 3.43* | 345.08*** | 0.109 | 3.99* |
| FA_6_ | 3.36 | 0.15 | 0.13 | 0.38 | 1.69 | 1.51 | 11.00** | 4.32* | 4.18* | 0.025 | 0.24 | 1.65 |
| FA_7_ |  |  |  |  |  |  |  |  |  | 0.00 | 0.14 | 0.15 |
| FA_8_ | 1.67 | 0.14 | 0.21 | 0.69 | 0.63 | 0.58 | 0.06 | 0.56 | 0.66 | 0.489 | 0.41 | 0.92 |
| FA_10_ | 0.28 | 1.25 | 1.18 | 10.98** | 0.30 | 0.23 | 5.87* | 3.88* | 3.84* | 341.70*** | 0.15 | 3.35* |

Degree of freedom for the error terms were 1854.

**Table A4** Descriptive statistics of leaf size ([*R* + *L*] / 2) and FA for plants at low (L), medium (M) and high (H) densities in infertile and fertile soil conditions, at three stages of day 30, 50 and 70. N is the number of individual values; significant values for the slope from regression of |R - L| versus leaf size were marked by *. *MS_m_* = measurement error mean square, *σ_i_^2^* = non-directional asymmetry, df = approximate degree of freedom for non-directional asymmetry after partitioning out measurement error.

| Stage  (days) | Density | N | (R+L)/2  Mean±SE | \|R-L\| vs. LS  Slope±SE | R - L | | | \|R-L\|=FA_1_  Mean±SE | FA_10_ | | |
| --- | --- | --- | --- | --- | --- | --- | --- | --- | --- | --- | --- |
|  |  |  |  |  | Mean±SE | Skew | Kurtosis |  | *MS_m_* | *σ_i_^2^* | df |
| Infertile soil | |  |  |  |  |  |  |  |  |  |  |
| 30 | L | 49 | 14.23±0.61 | 0.005±0.02 | 0.082±0.10 | -0.074±0.34 | -1.005±0.67 | 0.61±0.05 | 0.003 | 0.21 | 2.24 |
|  | M | 48 | 14.71±0.82 | 0.07±0.02* | 0.065±0.15 | -0.218±0.34 | -0.218±0.67 | 0.82±0.09 | 0.003 | 1.73 | 2.00 |
|  | H | 48 | 13.50±0.69 | 0.07±0.03* | -0.093±0.12 | 0.032±0.64 | 2.002±1.18 | 0.69±0.10 | 0.001 | 0.21 | 2.22 |
| 50 | L | 124 | 40.09±9.26 | 0.008±0.01 | 0.027±0.18 | 0.636±0.22 | 1.193±0.43 | 1.53±0.11 | 1.05 | 2.39 | 1.76 |
|  | M | 102 | 41.76±8.28 | 0.02±0.02 | 0.073±0.21 | -0.919±0.23 | 5.582±0.46 | 1.63±0.14 | 0.57 | 1.20 | 2.24 |
|  | H | 82 | 32.48±7.26 | 0.03±0.02 | 0.506±0.22* | 0.977±0.27 | 2.473±0.53* | 1.54±0.17 | 0.82 | 1.37 | 3.06 |
| 70 | L | 88 | 35.68±3.54 | 0.05±0.02* | -1.543±0.41* | 0.222±0.26 | -0.752±0.51* | 3.45±0.24 | 0.054 | 15.45 | 3.25 |
|  | M | 81 | 37.80±3.54 | 0.13±0.03* | -0.512±0.48 | 0.054±0.37 | -0.575±0.53 | 3.54±0.28 | 0.02 | 30.88 | 2.75 |
|  | H | 66 | 31.50±2.98 | 0.08±0.03* | 0.377±0.46 | -0.036±0.30 | -0.589±0.58 | 2.98±0.27 | 0.011 | 20.68 | 1.99 |
| Fertile soil | |  |  |  |  |  |  |  |  |  |  |
| 30 | L | 74 | 10.63±0.37 | 0.037±0.01* | -0.039±0.08 | -0.514±0.28 | 1.88±0.55 | 0.49±0.05 | 0.008 | 0.13 | 3.29 |
|  | M | 81 | 11.32±0.41 | 0.039±0.01* | 0.216±0.08* | -0.32±0.27 | 1.486±0.53* | 0.58±0.06 | 0.003 | 0.20 | 3.69 |
|  | H | 81 | 9.14±0.36 | 0.045±0.01* | 0.050±0.07 | 0.081±0.27 | -0.309±0.53 | 0.50±0.04 | 0.110 | 0.05 | 0.80 |
| 50 | L | 170 | 58.68±2.00 | 0.043±0.02* | -0.058±0.30 | -0.345±0.19 | 0.729±0.37 | 3.02±0.19 | 2.02 | 3.09 | 2.34 |
|  | M | 148 | 49.12±1.48 | 0.045±0.01* | 0.298±0.22 | 0.210±0.20 | 0.488±0.40 | 2.14±0.14 | 0.65 | 2.56 | 3.32 |
|  | H | 138 | 44.03±1.99 | 0.009±0.01 | 0.163±0.22 | 0.350±0.21 | 3.699±0.41 | 1.84±0.16 | 0.25 | 3.98 | 3.99 |
| 70 | L | 202 | 67.41±2.26 | 0.028±0.02 | 0.374±0.36 | 0.126±0.17 | -0.296±0.34 | 4.03±0.22 | 0.54 | 40.56 | 4.19 |
|  | M | 189 | 56.09±1.78 | 0.011±0.01 | 0.756±0.33* | 0.059±0.18 | 0.138±0.35* | 3.59±0.21 | 0.001 | 25.71 | 4.25 |
|  | H | 149 | 47.02±2.36 | 0.02±0.01* | -0.587±0.29* | -0.032±0.20 | -0.344±0.40* | 2.88±0.17 | 0.36 | 14.93 | 4.15 |

**Table A5** The results (mean squares [*MS*] and *F*-values) of two-way ANOVA for significance of all between-sides variation relative to measurement error, leaf side (S), individual (I), measurement (M) and side and individual interaction (S × I) on the width of leaves for plants grown in each block of low, medium and high densities in infertile soil conditions at three stages of day 30, 50 and 70.

| Density | Sources of variation | Day 30 |  |  |  | Day 50 |  |  |  | Day 70 |  |  |  |
| --- | --- | --- | --- | --- | --- | --- | --- | --- | --- | --- | --- | --- | --- |
|  |  | Df | *MS* | *F* | *p* | Df | *MS* | *F* | *p* | Df | *MS* | *F* | *p* |
| Infertile soil | |  |  |  |  |  |  |  |  |  |  |  |  |
| Low | Side | 1 | 0.58 | 0.083 | 0.77 | 11 | 0.088 | 0.003 | 0.96 | 1 | 168.42 | 4.76 | 0.03 |
|  | Leaf | 5 | 48.13 | 7.19 | <0.001 | 7 | 1602.06 | 49.12 | <0.001 | 7 | 4248.59 | 120.17 | <0.001 |
|  | Individual | 14 | 37.78 | 5.64 | <0.001 | 18 | 1086.17 | 33.30 | <0.001 | 17 | 1076.78 | 30.46 | <0.001 |
|  | Side × Individual | 14 | 0.43 | 0.064 | 1.000 | 18 | 2.88 | 0.088 | 1.000 | 17 | 35.73 | 1.01 | 0.45 |
|  | Measurement error | 1 | 0.001 | 0.000 | 0.99 | 1 | 0.009 | 0.000 | 0.99 | 1 | 0.005 | 0.000 | 0.99 |
| Medium | Side | 1 | 0.004 | 0.001 | 0.98 | 1 | 1.54 | 0.067 | 0.80 | 1 | 45.26 | 2.066 | 0.15 |
|  | Leaf | 4 | 206.15 | 31.22 | <.0.001 | 8 | 1164.08 | 50.74 | <0.001 | 6 | 3131.51 | 142.94 | <0.001 |
|  | Individual | 12 | 74.24 | 11.24 | <.0.001 | 17 | 895.97 | 39.05 | <0.001 | 16 | 782.47 | 35.85 | <0.001 |
|  | Side × Individual | 12 | 1.92 | 0.29 | 0.99 | 17 | 4.30 | 0.19 | 1.000 | 16 | 58.56 | 2.67 | 0.001 |
|  | Measurement error | 1 | 0.016 | 0.002 | 0.96 | 1 | 0.038 | 0.002 | 0.967 | 1 | 0.061 | 0.003 | 0.96 |
| High | Side | 1 | 0.405 | 0.052 | 0.82 | 1 | 10.98 | 0.66 | 0.42 | 1 | 0.052 | 0.004 | 0.95 |
|  | Leaf | 5 | 159.70 | 20.72 | <.0.001 | 5 | 1002.44 | 60.06 | <0.001 | 4 | 4568.39 | 314.65 | <0.001 |
|  | Individual | 13 | 46.330 | 6.01 | <.0.001 | 18 | 502.17 | 30.09 | <0.001 | 17 | 405.04 | 27.90 | <0.001 |
|  | Side × Individual | 13 | 0.48 | 0.06 | 1.00 | 18 | 5.14 | 0.31 | 0.997 | 17 | 34.54 | 2.38 | 0.002 |
|  | Measurement error | 1 | 0.00 | 0.00 | 0.996 | 1 | 0.00 | 0.00 | 0.999 | 1 | 0.008 | 0.001 | 0.98 |
| Fertile soil | |  |  |  |  |  |  |  |  |  |  |  |  |
| Low | Side | 1 | 0.20 | 0.14 | 0.71 | 1 | 14.01 | 0.44 | 0.51 | 1 | 15.14 | 0.43 | 0.51 |
|  | Leaf | 5 | 614.58 | 428.92 | <.001 | 9 | 1154.59 | 36.16 | <.001 | 11 | 240.21 | 6.87 | <.001 |
|  | Individual | 16 | 33.52 | 23.40 | <.001 | 17 | 2697.00 | 84.47 | <.001 | 17 | 3474.50 | 99.34 | <.001 |
|  | Side × Individual | 16 | 0.21 | 0.08 | 1.00 | 17 | 8.21 | 0.26 | 0.93 | 17 | 30.21 | 0.86 | 0.62 |
|  | Measurement error | 1 | 0.11 | 0.15 | 0.78 | 1 | 0.25 | 0.01 | 0.999 | 1 | 0.36 | 0.01 | 0.92 |
| Medium | Side | 1 | 3.78 | 0.99 | 0.32 | 1 | 16.65 | 0.40 | 0.53 | 1 | 113.71 | 1.72 | 0.19 |
|  | Leaf | 5 | 984.07 | 256.80 | <.001 | 9 | 2902.03 | 69.75 | <.001 | 12 | 2491.93 | 37.69 | <.001 |
|  | Individual | 15 | 30.70 | 15.84 | <.001 | 17 | 1768.22 | 42.50 | <.001 | 17 | 2892.70 | 43.75 | <.001 |
|  | Side × Individual | 15 | 0.40 | 0.11 | 1.00 | 17 | 5.76 | 0.14 | 1.00 | 17 | 51.43 | 0.78 | 0.72 |
|  | Measurement error | 1 | 0.003 | 0.00 | 0.98 | 1 | 0.65 | 0.02 | 0.90 | 1 | 0.001 | 0.00 | 0.997 |
| High | Side | 1 | 0.12 | 0.04 | 0.84 | 1 | 0.004 | 0.00 | 0.99 | 1 | 18.13 | 0.35 | 0.55 |
|  | Leaf | 5 | 684.37 | 235.96 | <.001 | 11 | 4549.31 | 88.43 | <.001 | 15 | 6835.60 | 132.66 | <.0001 |
|  | Individual | 14 | 43.94 | 15.15 | <.001 | 17 | 4184.13 | 81.33 | <.001 | 17 | 4573.05 | 88.75 | <.0001 |
|  | Side × Individual | 14 | 0.27 | 0.09 | 1.00 | 17 | 8.19 | 0.16 | 1.00 | 17 | 81.67 | 1.58 | 0.06 |
|  | Measurement error | 1 | 0.008 | 0.00 | 0.96 | 1 | 2.02 | 0.04 | 0.84 | 1 | 0.54 | 0.01 | 0.92 |


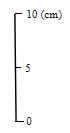


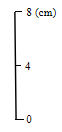


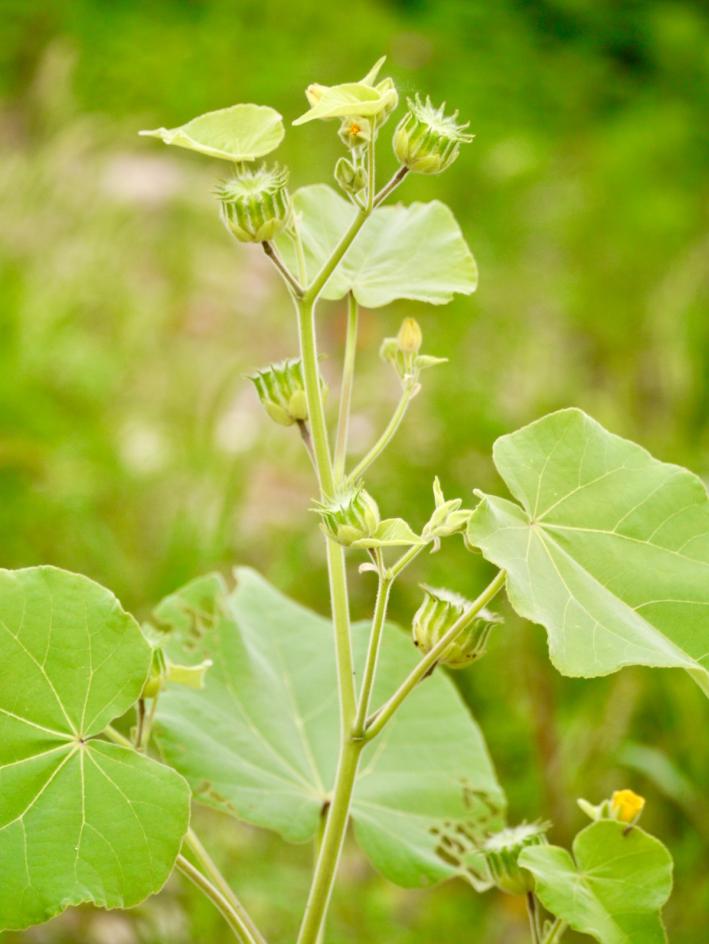

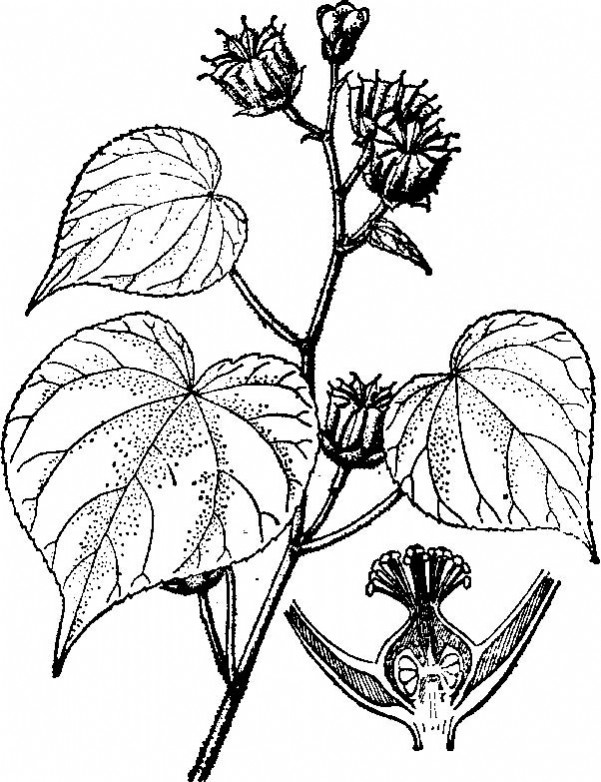


**d**

**b**

**c**

**a**

8 (cm)

8 (cm)


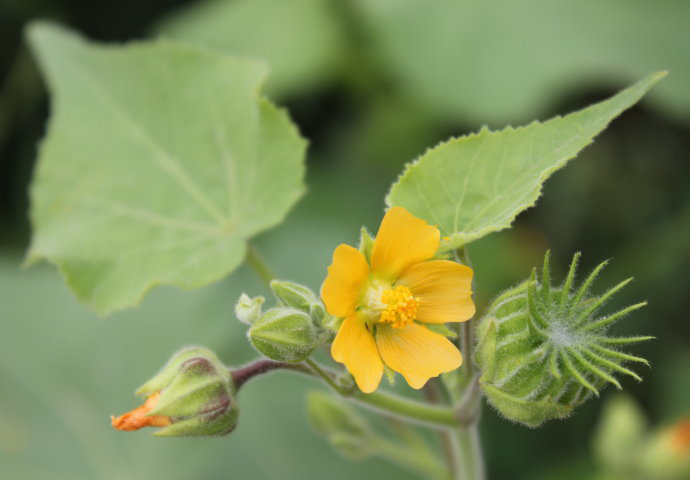

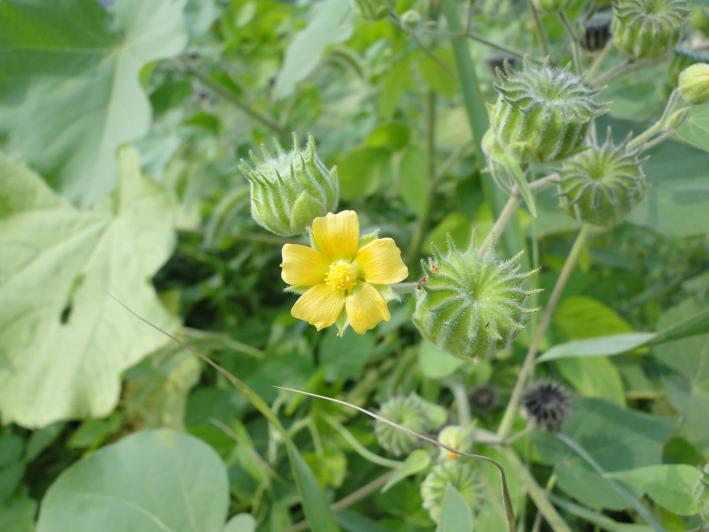


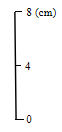


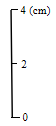


**Figure A1** The photographs (a, c, d) and sketch (b) for whole plant (a, b) and modules (c, d) of *Abutilon theophrasti*.

**Figure A2** Mean values (±SE) of all the other fluctuating asymmetry indexes (FA_3_-FA_8_) for leaf width in response to density, for plants under infertile (left) and fertile (right) soil conditions at day 30, 50 and 70 of plant growth. Different letters denote significant differences between density treatments within each of soil conditions and growth stage (LSD, *p* < 0.05), and *p*-values (from LSD) indicate differences between densities across all stages.
